# Low-Threshold, Multiple High-Order Harmonics Fiber Laser Employing Cr_2_Si_2_Te_6_ Saturable Absorber

**DOI:** 10.3390/nano13061038

**Published:** 2023-03-14

**Authors:** Nannan Xu, Xinxin Shang, Shuo Sun, Fuhao Yang, Weiyu Fan, Huanian Zhang, Dengwang Li

**Affiliations:** 1 Shandong Province Key Laboratory of Medical Physics and Image Processing Technology, School of Physics and Electronics, Shandong Normal University, Jinan 250014, China; 2 Shandong Provincial Key Laboratory of Optics and Photonic Device, School of Physics and Electronics, Shandong Normal University, Jinan 250014, China; 3 School of Physics and Optoelectronic Engineering, Shandong University of Technology, Zibo 255049, China

**Keywords:** low-threshold fiber laser, harmonic mode locking, 2D material

## Abstract

Abundant research findings have proved the value of two-dimensional (2D) materials in the study of nonlinear optics in fiber lasers. However, there remains two problems: how to reduce the start-up threshold, and how to improve the damage threshold, of fiber lasers based on 2D materials. A 15.1 mW low-threshold mode-locked fiber laser, based on a Cr_2_Si_2_Te_6_ saturable absorber (SA) prepared by the liquid-phase exfoliation method, is demonstrated successfully in this work. This provides a useful and economical method to produce SAs with low insertion loss and low saturation intensity. Besides, multiple high-order harmonics, from the fundamental frequency (12.6 MHz) to the 49th-order harmonic (617.6 MHz), mode-locked operations are recorded. The experimental results indicate the excellent potential of Cr_2_Si_2_Te_6_ as an optical modulator in exploring the soliton dynamics, harmonic mode locking, and other nonlinear effects in fiber lasers.

## 1. Introduction

Two-dimensional (2D) materials, with a van der Waals layered structure, possess attractive properties including quantum effects, microsize effects, and surface effects, for technical and practical applications such as in biomedicine, optical sensors, photodetectors, ultrafast photonics, and optical modulations [1,2,3,4,5,6,7,8]. Especially, distinctive physical singularities will occur as charge or heat transfer are determined on a plane, which has aroused the interest of researchers. One of the most active research directions is the preparation of 2D materials-based saturable absorbers (SAs) and their corresponding application in ultrafast fiber lasers [9,10,11,12]. This has great significance for the exploration of soliton dynamics, harmonic mode locking, and abundant other and complex nonlinear optical effects. The nonlinear saturable absorption effect is the basis of 2D materials used as SAs. The mechanism of saturation absorption of an SA is the Pauli blocking principle. When high-intensity light enters, the electrons in the 2D material will be excited from the valence band to the conduction band. After the conduction band is completely occupied, the photons will no longer be absorbed by the material, and SAs will show the saturable absorption property. Since the ultrafast fiber lasers realized by SAs have the advantages of simple structure and low cost [13], researchers have never stopped looking for more improved 2D materials, with better nonlinear effects and exceptional air stability, to realize excellent laser output.

There are two problems: (i) how to reduce the start-up threshold, and (ii) how to improve the damage threshold of fiber lasers based on 2D materials, that researchers are attempting to solve [9,14]. Among them, low-threshold fiber lasers have attracted great attention due to their advantages: a low mode-locked threshold means that ultrashort pulses can be obtained under a low operating voltage and current, corresponding to a low cost. Besides, a lower threshold leads to a lower intensity of spontaneous radiation noise and so the laser can operate with better stability. In addition, in the field of biomedicine, low-power lasers are needed, since proteins are vulnerable to damage in strong light fields. So low-threshold ultrafast lasers have great application potential. Currently, research on fiber lasers with a low threshold is developing rapidly [15,16,17]. The threshold concept arises due to the inevitable presence of self-radiating noise in fiber lasers [18], the magnitude of which can be reduced, but not eliminated completely. The advantages of low-threshold fiber lasers are also indirectly demonstrated by the direct relationship between the laser generation threshold and the laser emission. Therefore, it is important to study the factors affecting the start-up threshold, and to provide solutions. We find that, in addition to the self-radiating noise of fiber lasers, there are some crucial factors that affect the start-up threshold of 2D materials-based fiber lasers, which are, insertion loss and saturation intensity of 2D materials-based SAs. So, finding a suitable material, and optimizing the preparation method of SAs, is one of the ideas to reduce the start-up threshold.

Due to the continuous exploration in the field of materials, various materials have been applied to the research of low-threshold mode-locked fiber lasers, since graphene [19,20,21,22,23,24]. The availability of topological insulators (TIs) offers the possibility of making high-performance SAs [25,26,27]. Yin et al. prepared a Bi_2_Te_3_-based SA and output mode-locked pulses, with a pulse width of 1.26 ps, but the threshold power was still as high as 315 mW [28]. The appearance of black phosphorus (BP) provided researchers with more options to improve the performance of mode-locked fiber lasers [29,30]. Yu et al. prepared a BP-based SA, with a modulation depth of ~9.8%, and a mode-locked laser output with a pulse width of 1.58 ps was achieved, at a threshold power of 303 mW [31]. Later, transition metal dichalcogenides (TMDs) were extensively studied [32,33]. Lee et al. prepared a molybdenum diselenide (MoSe_2_) SA, by a liquid-phase exfoliation (LPE) method, and it was successfully applied in a fiber laser, with a threshold power of 274 mW [34]. Wu et al. achieved mode-locked operation in a fiber laser by using tungsten disulfide (WS_2_), at 1500 nm, for which the pump power reached 260 mW [35]. Some newly reported two-dimensional materials, such as single-element materials (Xenes) [36,37], and transition metal carbides or nitrides (MXenes) [38,39], were found to have great potential to realize low-threshold power mode-locked fiber lasers. This has created a new wave of interest among researchers in the search for new 2D materials with better properties.

Cr_2_Si_2_Te_6_, a new layered material belonging to the hexatellurosilicate family [40], has received a lot of attention in the fields of sensors [41,42] and optical devices [43,44]. The atomic layers of Cr_2_Si_2_Te_6_ are bound together by weak van der Waals forces, which also means that multilayered or few-layer 2D nanosheets can be obtained from their bulk-phase materials, by the liquid-phase exfoliation (LPE) method [45]. As a typical low-dimensional semiconductor material, the unique electronic, magnetic, and topological properties of Cr_2_Si_2_Te_6_ have been demonstrated by using first-principle calculations and simulations, based on density functional theory [46,47]. In addition, Cr_2_Si_2_Te_6_ has been used in a variety of applications, benefiting from its magnetic and electronic properties [48]. At the same time, Cr_2_Si_2_Te_6_ has an indirect band gap value of ~0.6 eV, which makes it suitable for applications in near-infrared lasers [49]. Moreover, the ferromagnetism property of Cr_2_Si_2_Te_6_ can exist not only in bulk, but also in single layers, which means that Cr_2_Si_2_Te_6_ may have the possibility to create robust and single-layer ferromagnetic insulators. Researchers are also continuing to explore its curious properties, such as thermoelectric effect, superconductivity, and photovoltaic effect. However, there are few studies on Cr_2_Si_2_Te_6_-based optical modulators, especially in low-threshold and harmonic mode-locked fiber lasers. In 2021, Zhu et al. reported a large-energy Er-doped fiber laser, with a Cr_2_Si_2_Te_6_-based saturable absorber, the threshold was over 200 mW and the frequency of mode-locked operation was only 1.61 MHz. In 2022, based on Cr_2_Si_2_Te_6_, Yang et al. reported a traditional soliton fiber laser whose threshold and frequency were 120 mW and 6.7 MHz.

In this work, a 15.1 mW low-threshold mode-locked fiber laser is demonstrated successfully. Such a low start-up threshold is mainly attributable to the low insertion loss and low saturation intensity of Cr_2_Si_2_Te_6_-based saturable absorber. The results indicate that the liquid-phase exfoliation (LPE) method is a useful and economical way to produce high-performance saturable absorbers. Besides, multiple high-order harmonics, from the fundamental frequency (12.6 MHz) to the 49th-order harmonic (617.6 MHz), mode-locked operations are recorded. All of these prove the excellent potential of Cr_2_Si_2_Te_6_ in exploring the soliton dynamics, harmonic mode locking, and other nonlinear optical effects in fiber lasers, as optical modulators.

## 2. Preparation and Characteristics of the Cr_2_Si_2_Te_6_-Based SA

**Figure 1:**
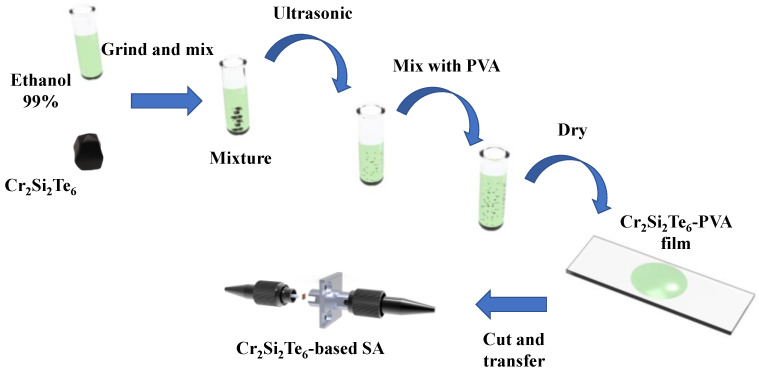
Preparation process of the Cr_2_Si_2_Te_6_-based SA.

The Cr_2_Si_2_Te_6_-based SA was prepared by the commonly adopted LPE method, considering the air stability property of Cr_2_Si_2_Te_6_. The preparation process is shown in Figure 1. Cr_2_Si_2_Te_6_ bulk (20 mg) was ground into powder and mixed with 50 mL 99% ethanol, in the first step. After 24 h of soaking, the mixture was placed in an ultrasonic cleaner for 48 h, in order to obtain Cr_2_Si_2_Te_6_ nanosheets. Then, 10 mL of the solution was taken and mixed with 10 mL 4 wt% polyvinyl alcohol (PVA) solution. This 20 mL solution was then put in the ultrasonic cleaner for 6 h. After this, the Cr_2_Si_2_Te_6_-PVA solution was pipetted onto a clean glass sheet. The glass sheet was then rotated at a constant speed. The solution formed a Cr_2_Si_2_Te_6_-PVA film after ~24 h of natural drying. Finally, the Cr_2_Si_2_Te_6_-based SA was prepared successfully by cutting the film into a sheet of appropriate size, and clamping the film between the end faces of two optical fiber patch cables.

**Figure 2:**
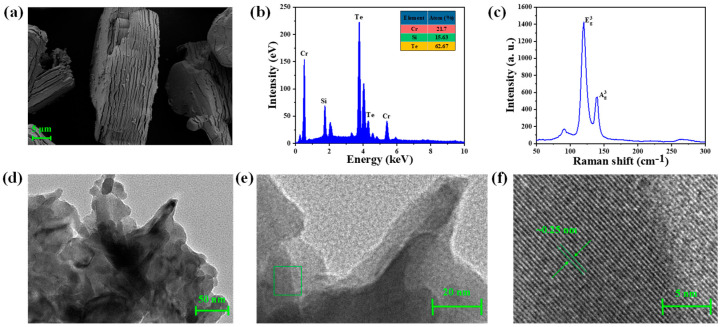
Characteristics of the Cr_2_Si_2_Te_6_ nanosheets. (**a**) SEM image, (**b**) EDS spectrum, (**c**) Raman spectrum, (**d**–**f**) HRTEM images at different resolutions.

**Figure 3:**
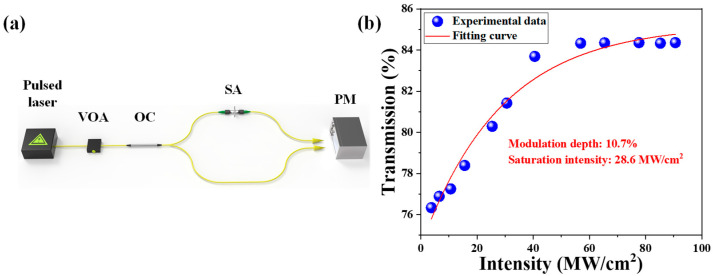
Nonlinear saturable absorption property of the Cr_2_Si_2_Te_6_-based SA. (**a**) The testing setup, (**b**) fitting curve.

In order to test the purity and physicochemical properties of the prepared Cr_2_Si_2_Te_6_ nanosheets, we characterized the material as follows. Figure 2a shows the picture from a scanning electron microscope (SEM), at a resolution of 5 μm. An obvious layered structure can be observed and the thickness of one Cr_2_Si_2_Te_6_ sheet is about 14 μm. The Cr_2_Si_2_Te_6_ spectrum from an energy dispersive spectrometer (EDS) is given in Figure 2b, in which the ratios of Cr, Si, and Te are 21.75%, 15.63%, and 62.67%, respectively, which correspond well with 1:1:3 in the chemical formula of Cr_2_Si_2_Te_6_. In addition, we used Raman spectroscopy to test the structural properties. Two strong Raman peaks were located at 120 cm^−^^1^, 140 cm^−^^1^, which correspond to the Eg3 and Ag3 modes of Cr_2_Si_2_Te_6_ [50]. The images from the high-resolution transmission electron microscope (HRTEM), shown in Figure 2d–f, exhibit an obvious layered structure, and clear crystal lattices, with a d-spacing of ~0.25 nm, can be observed, which indicates the prepared Cr_2_Si_2_Te_6_ nanosheets have excellent crystallinity properties. In addition, a femtosecond laser was used to test the nonlinear saturable absorption properties of the homemade SA. The testing setup is shown in Figure 3a. The central wavelength, pulse width, and frequency of the femtosecond laser are 1565 nm, 348 fs, and 10.8 MHz, respectively. A variable optical amplifier (VOA) is used to adjust the input pulse’s intensity. The input pulses are split into two beams through a 1:1 optical coupler (OC) and the SA is injected into one part of the OC. The output power of the two parts of the OC is recorded by a power meter (PM). The experimental data and fitting curve, fitted by

(1) $$T\left(I\right)=1-T_{\mathrm{ns}}-\Delta \;\exp\left(-\frac{I}{I_{\mathrm{sat}}}\right)$$

are shown in Figure 3b. T(I) is the transmission rate, T_ns_ is the non-saturable loss, ∆ is the modulation depth, I is the input intensity, and I_sat_ is the saturation intensity. From the formula, the saturation intensity, non-saturable loss, and modulation depth can be calculated, which are 28.6 MW/cm^2^, 15.78%, and 10.7%, respectively.

## 3. Results and Discussion

**Figure 4:**
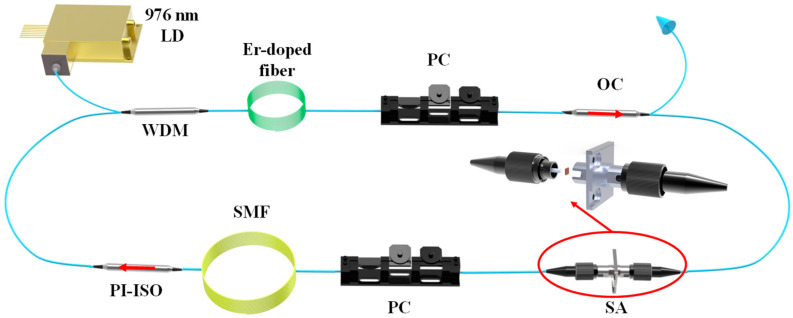
Structure of the low-threshold mode-locked fiber laser.

A low-threshold mode-locked fiber laser, based on a Cr_2_Si_2_Te_6_ SA, was constructed, with the structure shown in Figure 4. A 5 m Er-doped fiber (OFS-MP980) was utilized as the gain medium, which was pumped by a 976 nm laser diode (976 nm/600 mW) through a 1550 nm/980 nm wavelength division multiplexer (WDM). Two polarization controllers (PCs) were used to adjust the birefringence and polarization state of the cavity. Besides, a polarization-independent isolator (PI-ISO) was used to ensure the unidirectional transmission of the laser. A part of a single-mode fiber was inserted to control the dispersion, gain, and loss of the cavity. In order to realize mode-locked operation, the Cr_2_Si_2_Te_6_ SA was utilized as a mode locker. The operating condition of the fiber laser was recorded by an optical spectrum analyzer (Yokogawa, AQ6317B), a digital oscilloscope (Wavesurfer, 3054z) with a 2 GHz photo-detector, a radio frequency (RF) spectrum analyzer (Rohde & Schwarz, FPC1000), an auto-correlator (Femtochrome, FR-103XL), and an optical power meter through an optical coupler (OC, 10% output).

**Figure 5:**
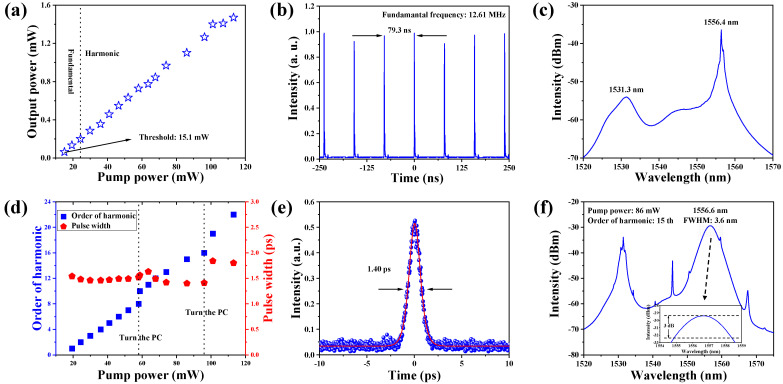
(**a**) Tendency of output power, (**b**) pulse trains under fundamental frequency, (**c**) optical spectrum under fundamental frequency, (**d**) trend of the order of harmonic and the pulse width changing with pump power, (**e**) auto-correlator trace of 15th harmonic pulse, (**f**) optical spectrum of 15th harmonic pulse.

**Figure 6:**
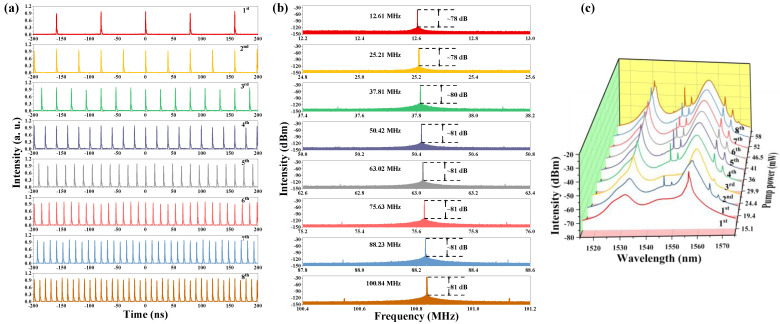
(**a**) Pulse trains from 1st–8th, (**b**) corresponding RF spectra, (**c**) changing trend of the optical spectra of the harmonic order from 1st–8th.

**Figure 7:**
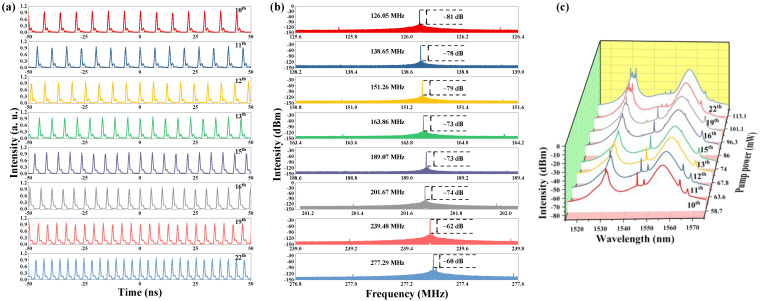
(**a**) Pulse trains from 10th–22nd, (**b**) corresponding RF spectra, (**c**) changing trend of the optical spectra of the harmonic order from 10th–22nd.

**Figure 8:**
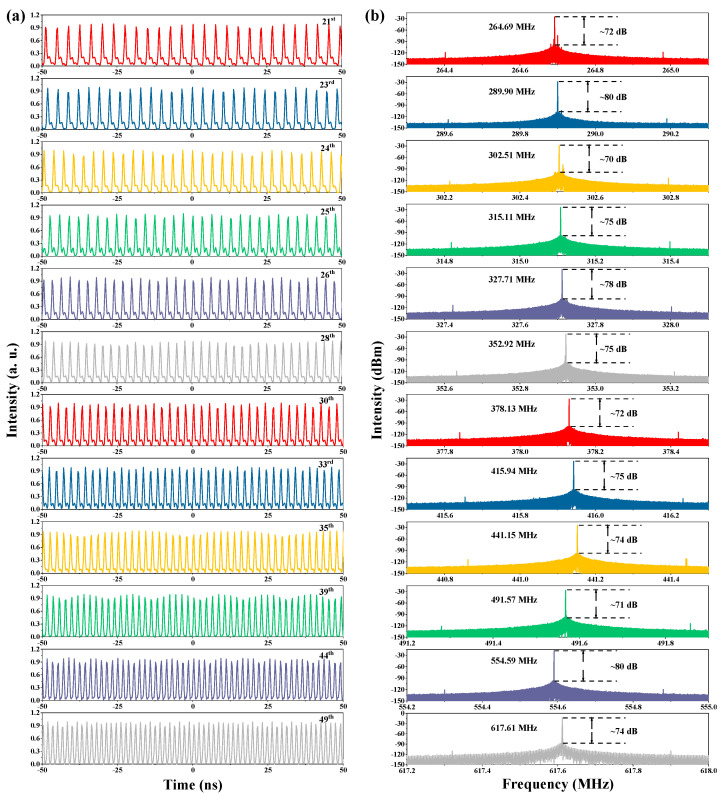
(**a**) Pulse trains from 21st–49th, (**b**) corresponding RF spectra.

**Figure 9:**
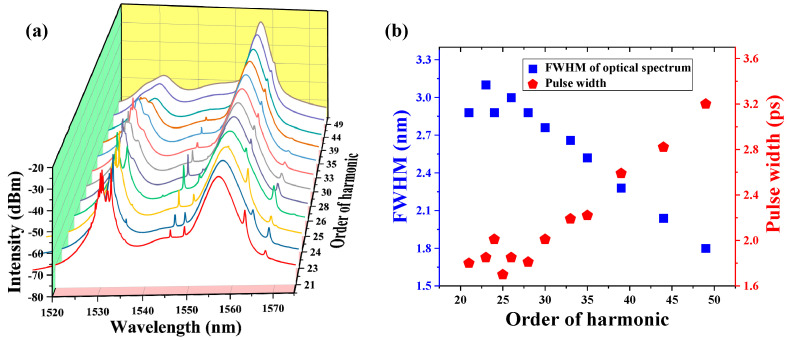
(**a**) Changing trend of the optical spectra of the harmonic order from 21st–49th, (**b**) changing trend of the FWHM and pulse width with the order of harmonics.

The output characteristics of the low-threshold mode-locked fiber laser are given in Figure 5, Figure 6, Figure 7, Figure 8 and Figure 9. Figure 5a shows the tendency of the output power. Mode-locked operation under the fundamental (1st) frequency can be observed, as the pump power is lower than 24.4 mW. When the pump power is larger than 24.4 mW, a stable harmonic mode-locked state is obtained, where the repulsive and attractive forces between pulses are balanced [51]. In our work, the threshold of the fiber laser is as low as 15.1 mW, which is mainly attributable to the low insertion loss and low saturable intensity of the Cr_2_Si_2_Te_6_ SA. Table 1 shows a comparison of mode-locked fiber lasers based on different 2D materials. The used Cr_2_Si_2_Te_6_ SA has a relatively large modulation depth and a smaller saturation intensity. Especially, the non-saturable loss is much lower than most other saturable absorbers. Benefiting from these excellent nonlinear properties of the homemade Cr_2_Si_2_Te_6_ SA, a lower start-up threshold and a higher signal-to-noise ratio (SNR) are realized in our work. The pulse trains and optical spectrum under the fundamental frequency (1st), are shown in Figure 5b,c. The fundamental frequency is 12.61 MHz, corresponding to the pulse interval of 79.3 ns. The center wavelength is located at 1556.4 nm, and there is a small peak caused by the continuous wavelength (CW) component, located at 1531.3 nm. Figure 5d exhibits the trend of the order of harmonic and the pulse width changing with pump power, in which the order of harmonic increases from the fundamental frequency to the 22nd harmonic, while the pulse width stays around 1.6 ps. Besides, the shortest pulse width recorded in our work was 1.4 ps, corresponding to the 15th harmonic, when the pump power was 86 mW. The auto-correlator trace of the 15th harmonic pulse is shown in Figure 5e, and the corresponding optical spectrum with the full width of half maximum (FWHM), of 3.6 nm, is given in Figure 5f.

Under a large pump power, multiple pulses will be aroused in the laser cavity, with appropriate net dispersion and strong nonlinear effects [65]. The attractive force between these multiple pulses will lead to the generation of soliton rain and bound state [66,67]. In contrast, the repulsive force between them will drive them away from each other, in a regular or irregular arrangement [51]. The generation of harmonic mode locking has been proved to be a result of the interaction of multiple pulses under gain depletion and recovery mechanism, the non-soliton component of radiation, and the acoustic wave effect [68,69,70]. In our work, when the pump power increases from 15.1 mW to 58 mW, the order of harmonic varies from 1st to 8th continuously. The pulse trains of the 1st–8th harmonics are given in Figure 6a. The corresponding RF spectra are exhibited in Figure 6b, in which the SNR is around 80 dB, which indicates that the fiber laser operates stably under both the fundamental frequency and harmonic states. Besides, the changing trend of optical spectra can be observed in Figure 6c. The intensity of the peak located at 1531.3 nm clearly increases with the increase in the pump power, and there is a tendency for it to split.

The pulse trains become a little unstable when the pump power is over 58 mW. So one of the PCs was turned slightly and we obtained the 10th harmonic pulse, under the pump power of 58.7 mW. The pulse trains of the 10th–22nd harmonics are given in Figure 7a, and the corresponding RF spectra are exhibited in Figure 7b. With the increase in the order, there is a marked decline in the SNR. But all of them are larger than 60 dB, which indicates the operating state of our fiber laser is stable enough. The optical spectra corresponding to the 10th–22nd harmonics can be observed in Figure 7c. The intensity of the peak of 1531.3 nm is equal to that of 1556.6 nm, and obvious splits can be observed in the higher-order harmonic. At the time, the Kelly sidebands have a trend of decline with the increase in the order of harmonics.

A higher order of harmonic pulses from the 21st to 49th can also be recorded by adjusting the PC, under the pump power of 113.1 mW. Figure 8a shows the pulse trains and Figure 8b gives the corresponding RF spectra. The SNR can be maintained between 70 dB and 80 dB, illustrating the stable operating state of the high-order harmonic. Different from the varying trend of the optical spectra, when the order is lower than the 22nd, the peak at 1531.3 nm changes in a completely opposite trend, with an increase in the order of the harmonic, which can be observed in Figure 9a. Besides, there is an obvious narrowing process of the FWHM of the optical spectra from 3.1 nm to 1.8 nm, which can be seen in Figure 9b. In addition, the pulse width increases from 1.7–3.2 ps with the increase in the order of harmonics.

When the pump power is higher than 113.1 mW, chaotic pulse generation can also be observed, but no matter how we adjust the PC, no stable pulse sequence is obtained. This is mainly limited by the deficiency of the intracavity nonlinear effect and dispersion [65].

## 4. Conclusions

A 15.1-mW low-threshold mode-locked fiber laser is demonstrated successfully in this work. Such a low start-up threshold is mainly attributable to the Cr_2_Si_2_Te_6_-based saturable absorber, which has low insertion loss and low saturation intensity. Besides, multiple high-order harmonics, from the fundamental frequency to 49th-order harmonic, mode-locked operations are recorded. The results indicate that the liquid-phase exfoliation method is a useful and economical way to produce high-performance saturable absorbers, and prove the excellent potential of Cr_2_Si_2_Te_6_ in exploring the soliton dynamics, harmonic mode locking, and other nonlinear optical effects in fiber lasers, as an optical modulator.

## Figures and Tables

**Table 1:** Comparison of threshold of 1.5 μm mode-locked fiber lasers based on different 2D materials.

Materials	Preparation Method	MD (%)	SI (MW/cm^2^)	NSL (%)	SNR (dB)	Threshold (mW)	Ref.
Graphene	CVD	2.7	-	-	64	47	[52]
	ME	3.6	0.08	44.2	70	38	[53]
Bi_2_Te_3_	LPE	4.8	-	73.4	60	80	[54]
Bi_2_Se_3_	Polyol	98	0.49 GW/cm^2^	-	-	65	[55]
Sb_2_Te_3_	LPE	3.9	106	87	74	44	[56]
BP	ME	0.6	-	-	65	80	[57]
	LPE	9	25	-	50	30	[58]
WS_2_	PLD	7.8	189	25.7	64	65	[59]
MoS_2_	MSD	19.48	4.137	38.53	75	40	[60]
WSe_2_	LPE	0.5	-	47.2	-	35	[61]
MoSe_2_	LPE	0.8	-	52	52	30.7	[62]
Ti_3_C_2_T_x_	LPE	0.96	256.9	73.5	71	146	[63]
Antimonene	LPE	9	1.3 GW/cm^2^	9	-	130	[36]
Bismuthene	LPE	2.03	30	82.5	55	153	[64]
Tellurene	LPE	5.06	34.3	58.6	55	85	[21]
Cr_2_Si_2_Te_6_	LPE	10.7	28.6	15.78	81	15.1	ours

MD: modulation depth, SI: saturation intensity, CVD: chemical vapor deposition, ME: mechanical stripping, PLD: pulse laser deposition, MSD: magnetron sputtering deposition, NSL: non-saturable loss.

## Data Availability

Data underlying the results presented in this paper are not publicly available at this time but may be obtained from the authors upon reasonable request.
